# USE OF AN ABDUCTION ORTHOSIS AFTER FEMORAL OSTEOTOMY IN CHILDREN WITH CEREBRAL PALSY

**DOI:** 10.1590/1413-785220263403e299980

**Published:** 2026-06-22

**Authors:** Lucas Cortizo Garcia, Fernando Cal Garcia, Ana Marta Pereira Medrado Faria, Larissa Landeiro Adorno, Antonio Gabriel Dias Passos, Vinicius Silva Rohrs

**Affiliations:** 1 Clinica Ortoped, Salvador, BA, Brazil.; 2 Hospital COT, Salvador, BA, Brazil.

**Keywords:** Cerebral Palsy, Osteotomy, Postoperative Period, Quality of Life, Paralisia Cerebral, Osteotomia, Período Pós-Operatório, Qualidade de Vida

## Abstract

**Objective:**

To evaluate the effectiveness and potential benefits of using customized abduction orthoses in the postoperative management of patients with cerebral palsy undergoing derotational osteotomy of the hip.

**Methods:**

A retrospective study was conducted with 14 patients (26 hips) with cerebral palsy and hip (sub)luxation, operated between 2021 and 2025 in private hospitals using the varus derotational femoral osteotomy technique. The analysis considered synthesis material positioning, achieved angle (Reimers’ Index), joint congruence on postoperative radiographs, and clinical outcomes recorded in medical charts after the use of the customized abduction orthosis.

**Results:**

Postoperative outcomes were satisfactory, with only 3.8% of hips presenting a new dislocation episode, requiring reoperation, and 7.6% showing superficial wound complications. All medical records reported comfort and practicality with orthosis use for both patients and caregivers.

**Conclusion:**

The use of customized abduction orthoses in the postoperative period of varus derotational femoral osteotomy in patients with cerebral palsy showed comparable effectiveness to traditional spica cast immobilization, while providing greater ease of home care and reducing the physical and emotional burden on caregivers. ***Level of Evidence II; Retrospective cohort study.***

## RESUMO

**Objetivo:**

Avaliar a eficácia e os benefícios do uso de órteses de abdução customizadas no pós-operatório de pacientes com paralisia cerebral submetidos à osteotomia derrotatória do quadril.

**Método:**

Estudo retrospectivo com 14 pacientes (26 quadris) com paralisia cerebral e subluxação do quadril, operados entre 2021 e 2025 em hospitais da rede privada pela técnica de osteotomia derrotatória varizante do fêmur. A análise considerou posicionamento do material de síntese, ângulo obtido (Índice de Reimers), congruência articular em radiografias pós-operatórias, além da evolução clínica registrada em prontuário após uso da órtese abdutora customizada.

**Resultados:**

Foram observados resultados pós-cirúrgicos satisfatórios, com apenas 3,8% dos quadris apresentando novo episódio de luxação, o que demandou reabordagem, e 7,6% registrando complicações superficiais de ferida operatória. Todos os prontuários relataram conforto e praticidade no uso da órtese, tanto para pacientes quanto para cuidadores.

**Conclusão:**

O uso da órtese abdutora customizada no pós-operatório da osteotomia derrotatória varizante do fêmur mostrou eficácia semelhante à imobilização tradicional com gesso spica, oferecendo maior facilidade nos cuidados domiciliares e reduzindo o impacto físico e emocional sobre os cuidadores. ***Nível de evidência II; Estudo de corte retrospectivo.***

## INTRODUCTION

Cerebral palsy (CP) is the leading cause of physical disability in childhood, affecting approximately 2-3 per 1,000 live births ^1^. Initially described by William Little in 1862, it is now characterized by permanent disorders of movement and posture development, causing limitations in motor activity, attributed to non-progressive changes occurring in the development of the fetal or infant brain^2^. Thus, it becomes a pathology that can lead to bone and joint deformities, with significant implications for the mobility and quality of life of patients.

**Figure 1:**
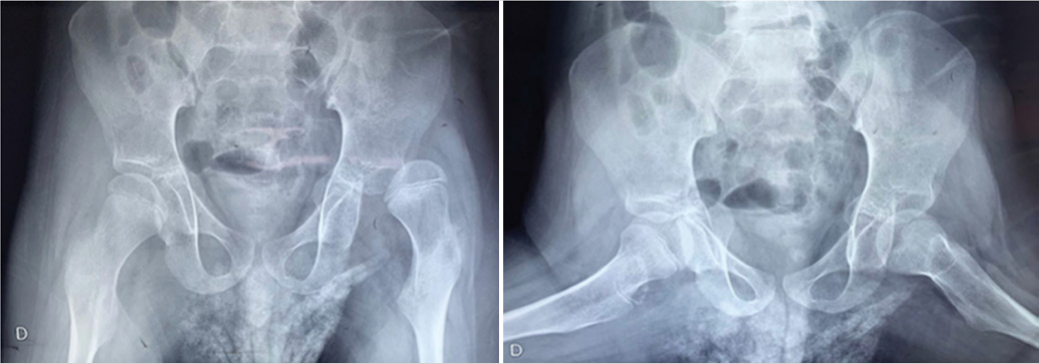
Preoperative pelvic radiographs in the AP and frog-leg views (left and right, respectively) of a child with cerebral palsy and left hip dislocation.

**Figure 2:**
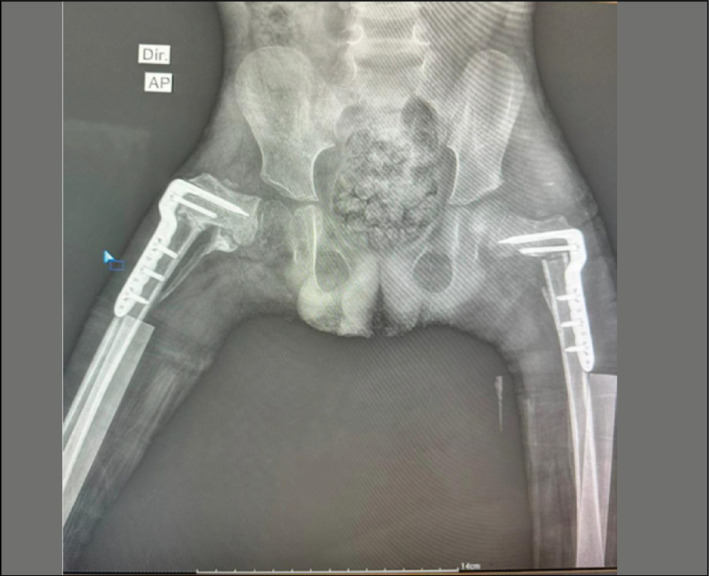
Postoperative follow-up radiograph of a child with cerebral palsy who underwent bilateral varus derotational femoral osteotomy.

One of the common symptoms in individuals with CP is muscle spasticity which, in the case of the hip, can culminate in a subluxation or even dislocation of the joint due to anteversion of the femoral neck and proximal femur valgus^3^ (Figure 1). The incidence of hip dysplasia in children with cerebral palsy varies significantly, being reported between 18% to 90% of patients, depending on the functional level according to the GMFCS (Gross Motor Function Classification System), where grades IV and V present a higher risk of developing hip subluxation and dislocation, with incidences that can reach 90%^4,5,6^. Therefore, surgical treatment of hip derotation osteotomy is indicated for those with indices suggesting unfavorable evolution, weighing the risks and benefits of the surgical procedure, aiming to relieve pain and improve locomotor function, potentially restoring the ability to ambulate and consequently the quality of life of the patient^7^ (Figure 2). Traditionally, postoperative immobilization after hip reconstruction in children with cerebral palsy has been performed using a spica cast, which encases the trunk and one or both legs, keeping the hip in an abducted position^8^. This method has been considered the gold standard for decades, aiming to protect the osteotomy during the bone healing process and prevent loss of surgical correction. However, the use of spica casts is associated with various complications and significant challenges for both patients and their caregivers.

**Figure 3:**
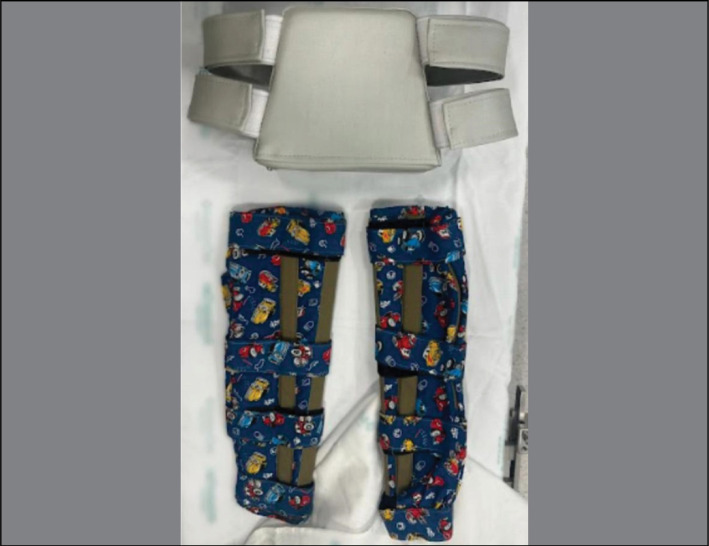
Custom abduction orthosis used in the study.

**Figure 4:**
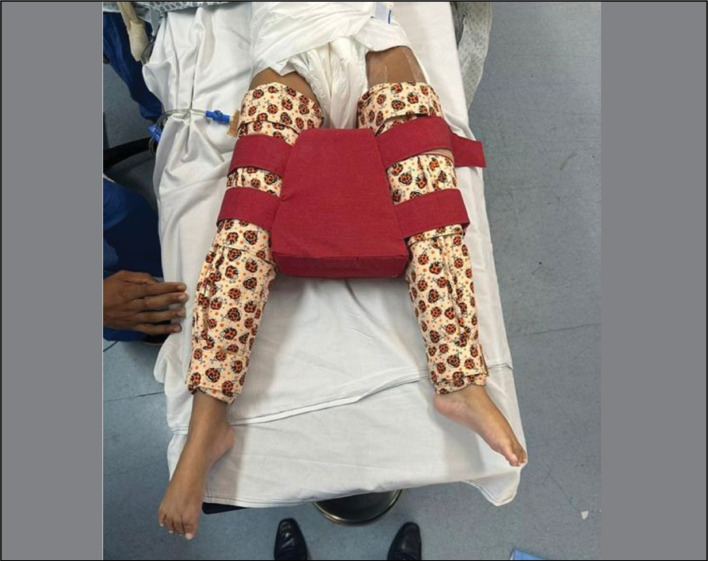
Patient using the custom-made abduction orthosis in the immediate postoperative period.

The proposed alternative to reduce such complications is the abductor orthosis, as used in this study, which consists of a triangular foam device with dimensions of 30-35 cm in length, a maximum width of 20-25 cm, a minimum width (at the bottom) of 10-15 cm, and adjustable height (Figure 3). This allows adjustment for different heights, with a range of up to 60 cm above the knee. To maintain knee extension, the orthosis is made of canvas with posterior duralumin stays and PVC sides, an elastic strap for knee compression, and a Velcro closure ranging from 20 to 80 cm, respecting the anatomical limits of 5 cm below the inguinal region and 3 cm above the malleolus (Figure 4).

The objective of this study is to evaluate the postoperative evolution in patients with cerebral palsy and hip subluxation who underwent hip derotation osteotomy, with special emphasis on the use of the customized abductor orthosis instead of plaster immobilization. The research will analyze the positioning of the locked synthesis material (plate and screws) in the radiographs following the procedure, as well as the acquired angulation and recovery of joint congruence. Thus, the evaluation of these criteria was made after the use of the orthosis, seeking to equate the results already identified in the literature with traditional immobilization approaches (spica cast). This study aims to highlight the effectiveness and potential benefits of using orthoses in postoperative management, contributing to the improvement of patient care and consequently the quality of life of both the patient and the caregiver during this period.

## MATERIALS AND METHODS

This is a retrospective study conducted through the analysis of medical records and follow-up radiographs of patients who underwent surgical procedures between 2021 and 2025, who used a customized abductor orthosis in the postoperative period. The research was conducted in an orthopedic clinic.

Patients with a diagnosis of cerebral palsy and hip subluxation or dislocation, who underwent hip derotation osteotomy during the established period, with documented postoperative follow-up, use of the customized abductor orthosis, and at least one follow-up consultation conducted within one year after the procedure, during which follow-up radiographs were evaluated, were included.

Patients whose medical records contained incomplete information about the postoperative period; those who did not undergo postoperative follow-up; patients who did not have follow-up radiographs after surgery; and those with severe comorbidities that could compromise the analysis of postoperative evolution of the hip, aiming to increase the reliability of the obtained data, were excluded from this study.

As this is a retrospective study, the risks to participants are minimal. All collected data was stored securely and anonymously, ensuring patient privacy in accordance with current ethical and legal standards. All patients included in the study signed the informed consent form.

## RESULTS

Among the sample of 20 patients diagnosed with cerebral palsy who underwent varus derotation femoral osteotomy, it was possible to include 14 patients (a total of 26 hips) with surgical approach dates between October 2021 and March 2025. The age of the patients at the time of surgery averaged 8.21 years, ranging from 5 to 14 years, with 6 female patients (42.8%) and 8 male patients (57.1%). The age of the patients at the time of surgery averaged 8.21 years, ranging from 5 to 14 years, with 6 female patients (42.8%) and 8 male patients (57.1%). This age distribution is consistent with the literature, which indicates that most hip reconstruction surgeries in cerebral palsy are performed in the first decade of life^9^.

Among the studied patients, the majority underwent the derotation osteotomy procedure on both hips (85.8%), with only 14.2% of the patients included in the study undergoing the procedure unilaterally. During postoperative follow-up, the custom abduction orthosis was used by all patients included in the study without exception (Table 1). During the follow-up (minimum of 2 months), only 1 recurrence of dislocation was observed (3.8% of the total hips studied), with a new approach made to correct the deformity, which progressed without further complications. This recurrence rate is within the acceptable parameters reported in the literature, where studies show recurrence rates ranging from 3.1% to 9.4%^10,11^.

**Table 1:** Demographic data of the study sample.

Variable	Data
Total number of patients evaluated	14 patients (26 hips)
Surgery period	October 2021 to March 2025
Average age at the time of surgery	8.21 years
Age range	5 to 14 years
Gender	6 females (42.8%) 8 males (57.1%)
Type of surgery performed	Bilateral: 12 patients (85.8%) Unilateral: 2 patients (14.2%)

In addition, 2 cases of superficial wound infection were identified (7.6%), which were treated with antibiotics without further repercussions or interference in the anatomical/mechanical outcome of the procedure. This rate of superficial infection is comparable to that reported by Amen et al., who found 2.7% of postoperative infections in their study^12^. (Table 2).

**Table 2:** Quantitative results of the study.

Variable	Data
Use of abduction orthosis in the postoperative period	100% of patients
Recurrence of dislocation	1 case (3.8% of hips)
Comparison with the literature	Recurrence rate consistent (literature: 3.1% to 9.4%)

Of the 14 cases analyzed, no other negative outcomes were found, such as loosening of the synthesis material, delayed consolidation, nonunion, osteomyelitis, or neurovascular changes. The absence of these more serious complications suggests that the abduction orthosis provided adequate protection during the critical period of bone healing.

An important finding of this study was that 100% of the medical records contained reports of satisfaction regarding comfort and practicality for the patient and caregiver concerning the use of the orthosis. This result corroborates studies that demonstrate greater satisfaction among caregivers with alternative immobilization methods^13,14^.

## DISCUSSION

Historically, postoperative immobilization of patients undergoing derotation osteotomy has been performed using spica casts, aiming to prevent loosening of the synthesis material and loss of the procedure due to the quality of osteoporotic bone, common in patients with cerebral palsy^15^. Segundo Vasconcellos et al. (2022) e Pisecky et al. (2022), the use of such immobilizations presents a rate of (sub)luxation in the postoperative period of 9.4% and 3.1%, respectively^16,17^.

In the present study, the dislocation recurrence rate was 3.8% (1 case in 26 hips), which is lower than the results reported in the literature for spica cast immobilization. This finding is particularly significant as it demonstrates that the use of abduction orthoses does not compromise the effectiveness of treatment in terms of maintaining the surgical correction achieved.

This study shows that the use of customized abduction orthoses in the postoperative period of varus derotation femoral osteotomy in children with cerebral palsy has an effectiveness equivalent to the traditional method of spica cast immobilization, with significant advantages in terms of comfort, practicality, and quality of life for both patients and their caregivers.

The study by Vasconcellos et al., which included 233 children (436 VDROs) directly comparing spica cast versus abduction orthosis, found no statistically significant differences in the radiographic parameters (aMPFA, AI, MP) after one year of follow-up ^17^. These findings corroborate the results of the present study and reinforce the evidence that abduction orthoses are a safe and effective alternative to traditional spica cast.

One of the main advantages of abduction orthoses over spica cast lies in the ease of maintaining personal hygiene and skin care. The cast, due to its rigid and non-removable nature, creates an environment conducive to the accumulation of moisture, dirt, and organic debris, complicating hygiene care ^18^. This limitation is problematic in children with cerebral palsy, who often experience urinary and fecal incontinence, increasing the risk of contamination and infections ^19^.

Pressure ulcers represent a serious and potentially severe complication in immobilized patients ^20^. The spica cast, due to its rigidity and inability to adjust after application, can create excessive pressure points on bony prominences, leading to this complication ^21^.

In contrast, abduction orthoses allow full access to high-risk areas for the development of pressure ulcers, facilitating daily hygiene and allowing regular skin inspection. The material used in the fabrication of the orthoses, typically viscoelastic foam or similar materials, offers better pressure distribution ^22^.

In the present study, no significant skin complications related to the use of the abduction orthosis were observed, which may be attributed to the ease of cleaning and the possibility of temporarily removing the device for specific care. This finding favorably contrasts with the rates of skin complications reported for spica cast in the literature.

The impact of the immobilization method on the quality of life of caregivers is an often underestimated aspect, but it is extremely important in the choice of postoperative treatment^23^. Children with cerebral palsy often require intensive and specialized care, and the addition of a spica cast can significantly increase the workload and stress of caregivers, as it demands logistics that include difficulties in transportation, the need for special equipment, and limitations in daily life^24^.

Studies on caregivers’ perceptions of orthotic devices demonstrate that ease of use and practicality are determining factors in treatment adherence^25^.

Additionally, patient comfort is a fundamental aspect that directly influences quality of life during the postoperative immobilization period^26^. The spica cast, due to its rigidity and weight, often causes significant discomfort, especially in children with cerebral palsy who may exhibit spasticity and involuntary movements.

Abduction orthoses allow for adjustment of pressure and positioning to optimize individual comfort for each patient, which further promotes treatment adherence with less need for analgesia and better sleep quality^26^. In the present study, 100% of the records contained reports of satisfaction regarding comfort, a finding that corroborates the literature on the advantages of orthoses in terms of patient comfort.

Although evaluating economic aspects was not a specific objective of this study, it is important to consider the financial implications of different immobilization methods. The spica cast, despite having a seemingly lower initial cost, can generate significant additional costs related to complications and special care needs^27^.

The skin complications associated with the spica cast may require additional medical treatment, including unscheduled consultations, medication use, and in severe cases, surgical procedures for the treatment of pressure ulcers. Additionally, the need for special equipment for the transport and care of children with spica casts may represent additional costs for families.

Shirley et al. demonstrated that alternative immobilization methods, including orthoses, can be cost-effective when considering the total treatment costs, including complications and additional care needs^27^. The previously mentioned reasons are factors that contribute to the cost-effectiveness of orthoses.

It is also worth noting that this study presents some limitations that should be considered in the interpretation of the results. First, this is a retrospective study with a relatively small sample (14 patients, 26 hips), which may limit the generalization of the findings.

Second, the assessment of patient and caregiver satisfaction was based on medical records, which may not fully capture the subjective experience of the participants. Future studies using validated instruments for quality of life and satisfaction assessment could provide more robust data on these aspects^28^.

Finally, the absence of a contemporary control group using spica casts limits the ability for direct comparison. Although the results were compared with data from the literature, variations in surgical techniques, patient characteristics, and evaluation criteria may influence these comparisons.

On the other hand, the findings of this study have important implications for clinical practice. The demonstration that customized abduction orthoses can offer clinical outcomes equivalent to the spica cast, with significant advantages in terms of comfort, financial practicality, and quality of life, suggests that this method should be considered as a first-line treatment for postoperative immobilization in children with cerebral palsy undergoing femoral derotation osteotomy.

## CONCLUSION

The data obtained showed that the use of the customized abduction orthosis in the postoperative period of the varus derotation osteotomy of the femur in patients with cerebral palsy presents satisfactory radiographic results, with adequate maintenance of surgical correction and joint congruence equivalent to the use of the traditional immobilization method (spica cast).

The observed dislocation recurrence rate (3.8%) is within the acceptable parameters reported in the literature for traditional immobilization methods, demonstrating that the abduction orthosis does not compromise the treatment's effectiveness in terms of maintaining the surgical correction achieved.

In light of the comprehensive analysis of the collected data and the methodology of the study in question, it was identified that the abduction orthosis presents itself as a safe and effective alternative, bringing significant clinical, practical, social, and financial benefits compared to the traditional spica cast, without compromising the surgical outcome.

It is recommended that customized abduction orthoses be considered as a first-line method for postoperative immobilization in this patient population, with the need for more randomized prospective studies with larger samples and longer follow-up to confirm these findings and establish evidence-based guidelines for clinical practice.

## Data Availability

The authors confirm that the data supporting the findings of this study are available in the article itself. Additionally, the datasets used and/or analyzed during the current study are available from the corresponding author upon reasonable request.
